# Differential Susceptibility to Propofol and Ketamine in Primary Cultures of Young and Senesced Astrocytes

**DOI:** 10.1155/2024/8876548

**Published:** 2024-04-10

**Authors:** Liang Huang, Ferit Tuzer, Abigail Murtha, Michael Green, Claudio Torres, Henry Liu, Shadi Malaeb

**Affiliations:** ^1^Department of Anesthesiology and Perioperative Medicine, New York University Langone Health, Grossman School of Medicine, New York, NY, USA; ^2^Department of Neurobiology and Anatomy, Drexel University College of Medicine, Philadelphia, PA, USA; ^3^Department of Pediatrics, Drexel University College of Medicine, Philadelphia, PA, USA; ^4^Department of Anesthesiology and Perioperative Medicine, Thomas Jefferson University, Sidney Kimmel Medical College, Philadelphia, PA, USA; ^5^Department of Anesthesiology and Critical Care, Perelman School of Medicine, Philadelphia, PA, USA

## Abstract

The adverse effects of general anesthesia on the long-term cognition of young children and senior adults have become of concern in recent years. Previously, mechanistic and pathogenic investigations focused on neurons, and little is known about the effect of commonly used intravenous anesthetics such as propofol and ketamine on astrocytes. Recently, astrocyte dysfunction has been implicated in a wide range of age-related brain diseases. In this study, we examined the survival and viability of both young and senescent astrocytes in culture after adding propofol and ketamine to the media at varying strengths. Oxidative stimulus was applied to commercially available fetal cell lines of human astrocytes in vitro to induce morphological changes in cellular senescence. Our results indicate that propofol reduces the survival of young astrocytes as compared to controls, as well as to ketamine. These effects were seen in comparisons of total cell count and at both high and low dose concentrations. High doses of propofol also significantly reduced cell viability compared to those exposed to baseline controls and ketamine. Senescent astrocytes, on the other hand, demonstrated cell count reductions as compared to baseline controls and ketamine when exposed to either DMSO or propofol. The data show differential susceptibility of young astrocytes to propofol than to ketamine. The observed cell count reduction may be related to the adverse effects of propofol on mitochondrial function and free radical production, as described in previous studies. We speculate that ketamine may have a more favorable safety profile in infants and young children.

## 1. Introduction

Astrocytes, the most common glial cell in the central nervous system, play multiple functional roles in brain physiology, including maintaining neuronal function, providing growth factors and metabolic support to neurons, glutamate shuttling, regulating neurotransmitter release, water and electrolyte homeostasis, and maintaining the blood-brain barrier [1–4]. Over time, there has been an increased understanding of how astrocytic dysfunction contributes to overall brain pathology. Studies have indicated the significance of astrocytes in normal processes, such as aging, as well as in abnormal development and degeneration of the brain. These studies have described the role of astrocytes in the induction, propagation, and resolution of neurodegenerative disease [4]. Astrocytes have also been shown to function as both perpetrators and protectors in the pathology of cerebral ischemia, major depressive disorder, stroke, traumatic brain injury, and dementia [5–9].

Anesthesia often involves exposure to volatile anesthetic gases, as well as the administration of intravenous agents such as propofol and ketamine. Propofol [2,6-diisopropylphenol] is frequently used for procedural sedation and general anesthesia in children and adults [10]. Ketamine [2-(o-chlorophenyl)-2-(methylamino) cyclohexanone hydrochloride] is commonly used in pediatric patients for procedures [11, 12]. These medications are often chosen because of their rapid effect, short action, and low side effect profile.

Recent advances in anesthesia have led to increased survival of vulnerable populations at the extremes of age, both in the very young and premature as well as in the very old. These individuals present with an increased risk of adverse outcomes, including neurocognitive impairment after anesthesia [13–16]. Extensive population-based cohort studies suggest that adult patients who underwent anesthesia and surgery had double the risk of dementia and were diagnosed in a shorter amount of time [17]. Clinical studies have also demonstrated the adverse effects of anesthetics on neurodevelopment throughout early life. A study of pediatric patients with repeated anesthetic exposure demonstrated a decreased average processing speed and parental report of executive function, fine motor skills, behavior, and reading ability [18]. Another postmortem study of pediatric patients who had undergone anesthesia also showed higher rates of reactive gliosis [19].

However, studies on astrocytes and anesthesia have been limited [20], and there has been a paucity of research investigating the effects of anesthesia on astrocyte viability. Therefore, the present study examines the susceptibility of young and senescent astrocytes in primary cultures to commonly used intravenous anesthetics such as propofol and ketamine.

## 2. Methods

### 2.1. Cell Culture and Senescence Induction

Human fetal astrocytes (passage 1) were obtained from ScienCell Research Laboratories (cat.# 1801; Carlsbad, CA, USA) and were grown at 37°C, 5% CO_2_ in an aqueous medium supplemented with 2% fetal bovine serum (FBS), growth supplement, and penicillin/streptomycin as previously described [21]. The cells were seeded at a standard density of 1 × 10^4^ cells/cm^2^ and cultured until 70%–80% confluence was reached. After each passage, the cells were trypsinized and counted. The cumulative population doubling (PD) level was then calculated as previously described [8, 21] using the formula NH/NI = 2^*x*^, where NH represents the number of cells at harvest, NI represents the initial number of seeded cells or inoculum, and *x* is the number of population doublings.

Cells were considered to be early passage (young cells) when less than 50% of their replicative life span was completed within PD <10. Cells were used at PD 7.2 for the young astrocyte experiments. Oxidative stress was applied to induce premature senescence, mimicking an aged population of cells as previously described [21, 22]. Cells were again seeded at 1 × 10^4^ cells/cm^2^ and then treated with 200 *μ*M hydrogen peroxide (H_2_O_2_) the following day for two hours. Senescence was reached at least five days after starting treatment [21]. Cells were harvested seven days after treatment and assayed for senescence, characterized by the flattened and enlarged morphology and by the cessation of division.

### 2.2. Administration of Propofol and Ketamine

Equivalent aliquots of cell suspensions were randomly assigned to the study groups. Propofol (Fresenius Kabi, Lake Zurich, IL 60047) was emulsified with dimethyl sulfoxide (DMSO) as a vehicle compound to make a stock solution of 15 mM propofol in 74% (v/v) DMSO. The concentrations of propofol used were 30 *μ*M (low-dose condition), 100 *μ*M (medium-dose condition), and 300 *μ*M (high-dose condition). These concentrations were obtained by adding 1.9, 6.4, and 19 *μ*L of the 15 mM propofol to the media for a total volume of 950 *μ*l. DMSO alone was added to culture media in a total volume of 950 µL at the above ratios (1.9, 6.4, and 19 *μ*L), yielding 1% (low-dose condition), 3.34% (medium-dose condition), and 10% v/v (high-dose condition) DMSO as corresponding vehicle controls for propofol. Ketamine (Sigma, St. Louis, MO) was added to culture media at concentrations of either 30 *μ*M (low-dose condition) or 300 *μ*M (high-dose condition). The concentrations of propofol and ketamine used corresponded to the range of plasma concentrations observed in patients anesthetized with these agents [23, 24]. In addition, cultures of cells without anesthetic treatment were used as baseline controls.

The cells were incubated at 37°C for up to seven hours with DMSO alone, propofol + DMSO, or ketamine at the final concentrations indicated in the astrocyte medium described above. After incubation, the culture media containing floating cells were collected, and attached cells were washed once with 1 mL Dulbecco-modified phosphate‐buffered saline (PBS, with calcium and magnesium) and then resuspended in 100 *μ*l 0.25% trypsin at 37°C for three minutes. The culture media were added back to the cells, and the cellular suspensions were pipetted five to six times to disperse the cells homogeneously. Cell counts were measured by flow cytometry, whereas cell viability was assessed by cell viability assay.

### 2.3. Cell Viability Assay

The ViaCount Assay (EMD Millipore, Burlington, MA) was used to distinguish the viability of cells based on differential permeabilities of two DNA-binding dyes in the Guava ViaCount® reagent. A nuclear dye was used to stain only cells with a nucleus, whereas a viability dye stains dying cells. Altogether, this combination of dyes differentiates between viable and dead cells. Debris was excluded from results based on negative staining with the nuclear dye and by predetermined size cutoff in flow cytometry. A volume of 50 *μ*L of cell suspension was mixed with 250 *μ*L of Guava ViaCount reagent (fivefold dilution) in a 1.5-mL microcentrifuge tube per the manufacturer's recommendations. Aliquots (71 *μ*L) were collected from each sample at a concentration of two to ten cells/*μ*L, corresponding to 150 to 800 cells per measurement volume. Samples were mixed well, incubated for about five minutes at room temperature, and kept protected from light. All treatments were made in triplicate wells. ViaCount measurements were made on a Guava EasyCyte system (Millipore). Cells were kept on ice while waiting for measurement.

### 2.4. Statistical Analysis

The baseline control astrocytes grown in media without additives were evaluated for cell count. These cells were obtained from the same batch and cultured on the same multi-well culture plate of each set of experiments, simultaneously with the other experimental groups. For relative quantification of cell growth, the well showing the best growth of astrocytes in control media was defined to be 100% and referred to as baseline control. The data from the experimental groups were presented as the means of % best growth, 95% confidence intervals of the baseline control in the wells run on the same day of the experiment. One-way ANOVA with Bonferroni correction for post hoc comparisons was used. All analyses utilized SigmaPlot 13.0 (Systat Software, Inc. San Jose, CA). Statistical significance was determined at *p*  <  0.05.

## 3. Results

### 3.1. Effects of Senescence on Astrocytes' Growth in Culture

**Figure 1:**
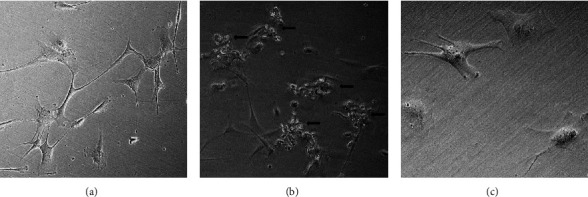
Representative cell viability assay of primary astrocytes. Representative phase-contrast microscopic images (10X) showing young human fetal astrocytes in primary cultures without any additives (a) and dying and detaching young astrocytes in cultures containing 300 *μ*M propofol in DMSO ((b), arrows). (c) Senescent human astrocytes show morphological changes indicative of cellular senescence, including flattened cell bodies and the presence of cytoplasmic vacuoles.

Overall, 99.1 ± 1.7% of young astrocytes (*N* = 375 ± 56 cells) in 71 *μ*L measurement volume remained viable after incubation in culture media with no vehicle or drug for seven hours, compared to 87.3 ± 20.5% of senescent cells (*N* = 349 ± 41 cells; *p* = N.S. vs. young). Senescent human astrocytes showed morphological changes indicative of cellular senescence, including flattened cell bodies and the presence of cytoplasmic vacuoles compared to young cells (Figure 1).

### 3.2. Young Cells

#### 3.2.1. Effects of Different Media on Young Astrocyte Growth in Primary Culture

Young astrocytes were incubated with control media, DMSO, propofol + DMSO, and ketamine for seven hours and were then analyzed based on resultant total cell count, dose-response cell count, and total viability assays.

#### 3.2.2. Total Cell Count

**Figure 2:**
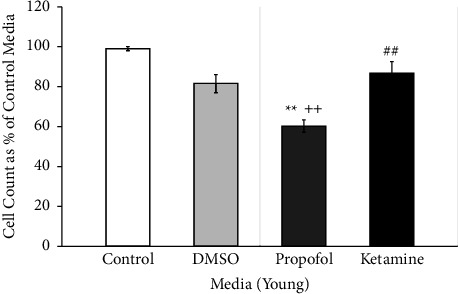
Cell counts of young astrocytes in DMSO, propofol, and ketamine. The average number of young astrocytes per exposure at all doses after seven hours of incubation in different media was expressed as a percentage of the best growth in control media for each set of experiments and presented as means, 95% confidence intervals. Control (*N* = 4; white bar), DMSO (*N* = 12 with low, medium, and high preparations included; light gray bar), propofol + DMSO (*N* = 12 with low, medium, and high preparations included; dark gray bar), and ketamine (*N* = 6 with low and high preparations included; closed bar). ^*∗∗*^*p* < 0.01 vs control; ^++^*p* < 0.01 vs DMSO; ^##^*p* < 0.01 vs propofol + DMSO. ANOVA with Bonferroni correction for post hoc comparisons.

Cell counts of young astrocytes were combined between all doses of each assigned medium. The effects of DMSO, propofol + DMSO, and ketamine on young primary culture human astrocytes are shown in Figure 2. Baseline control media demonstrated a total cell count of 375 ± 56 cells per aliquot. Astrocytes incubated for seven hours in propofol dissolved with DMSO saw a 39.2% reduction of total cell count as opposed to the baseline control, which is the greatest reduction in total cell count as compared to either DMSO vehicle alone or ketamine (*p* < 0.01). Propofol dissolved in DMSO also had a significant 26% cell count reduction versus DMSO as well as a 29% reduction versus ketamine (both at *p* < 0.01). There was no significant difference in total cell count between the baseline control group and either DMSO or ketamine.

#### 3.2.3. Dose-Response Cell Count

**Figure 3:**
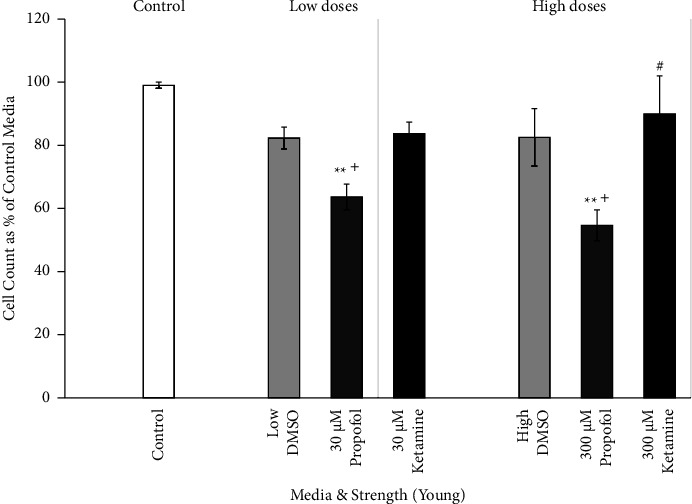
Cell count dose response of young astrocytes to DMSO, propofol, and ketamine. The average number of young astrocytes per dose after seven hours of incubation in escalating strengths of different media (30 *μ*M, low; 300 *μ*M, high; 100 *μ*M of DMSO and propofol + DMSO not shown), expressed as percent of best growth in control media for each set of experiments and presented as means, 95% confidence intervals. Control (*N* = 4; white bar), low DMSO corresponding to 74% DMSO used to prepare 30 *μ*M propofol (*N* = 3; light gray bar, left), high DMSO corresponding to 74% DMSO used to prepare 300 *μ*M propofol (*N* = 6; light gray bar, right), low propofol + DMSO (*N* = 3; 30 *μ*M dark gray bar, left), high propofol + DMSO (*N* = 6; 300 *μ*M dark gray bar, right), low ketamine (*N* = 3; 30 *μ*M closed bar, left), and high ketamine (*N* = 3; 300 *μ*M closed bar, right). ^∗∗^*p* < 0.01 vs control; ^+^*p* < 0.05 vs DMSO; ^#^*p* < 0.05 vs propofol + DMSO. ANOVA with Bonferroni correction for post hoc comparisons.

Dose-response cell counts of young astrocytes were examined between the low and high doses of each medium type. The effects of DMSO, propofol + DMSO, and ketamine on young primary culture human astrocytes are shown in Figure 3. Dose-response studies demonstrated variable effects of medication on cell count reductions. Low doses of incubation media were set at 30 *μ*M. Propofol dissolved in DMSO demonstrated significant cell count reductions versus the baseline control (*p* < 0.01) and versus DMSO alone (*p* < 0.05). High doses corresponded with the administration of 300 *μ*M of media. Once more, propofol dissolved in DMSO had a significant cell count reduction versus the baseline control (*p* < 0.01) and versus DMSO alone (*p* < 0.05). In the high-dose condition, propofol dissolved in DMSO also showed a significant cell count reduction compared to ketamine (*p* < 0.05). There were no significant differences between the baseline control and DMSO alone or ketamine, nor between DMSO alone and ketamine at either concentration.

#### 3.2.4. Viability

**Figure 4:**
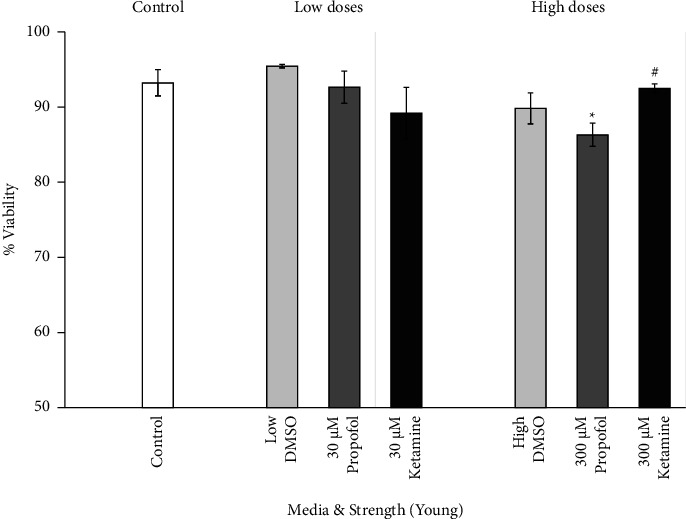
Viability dose response of young astrocytes to DMSO, propofol, and ketamine. Average percent viability of young astrocytes per dose after seven hours of incubation in escalating strengths of different media (30 *μ*M, low; 300 *μ*M, high; 100 *μ*M of DMSO and propofol + DMSO not shown), presented as means, 95% confidence intervals. Control (*N* = 4; white bar), low DMSO corresponding to 74% DMSO used to prepare 30 *μ*M propofol (*N* = 3; light gray bar, left), high DMSO corresponding to 74% DMSO used to prepare 300 *μ*M propofol (*N* = 6; light gray bar, right), low propofol + DMSO (*N* = 3; 30 *μ*M dark gray bar, left), high propofol + DMSO (*N* = 6; 300 *μ*M dark gray bar, right), low ketamine (*N* = 3; 30 *μ*M closed bar, left), and high ketamine (*N* = 3; 300 *μ*M closed bar, right). ^∗^*p* < 0.05 vs. control; ^#^*p* < 0.05 vs. propofol + DMSO. ANOVA with Bonferroni correction for post hoc comparisons.

The viability of young astrocytes was examined between high and low doses of the different medium types. The effects of DMSO, propofol + DMSO, and ketamine on young primary culture human astrocytes are shown in Figure 4. Assessment of dose-response viability demonstrated significant effects only in high doses of propofol dissolved in DMSO versus the baseline control (*p* < 0.05) and versus high-dose ketamine (*p* < 0.05). No other significant reductions in viability were noted.

### 3.3. Senesced Cells

#### 3.3.1. Effects of Different Media on Senescent Astrocyte Growth in Primary Culture

Senesced astrocytes were incubated with control media, DMSO, propofol dissolved in DMSO, and ketamine. After seven hours, they were then analyzed based on the resultant total cell count, dose-response cell count, and total viability assays.

#### 3.3.2. Total Cell Count

**Figure 5:**
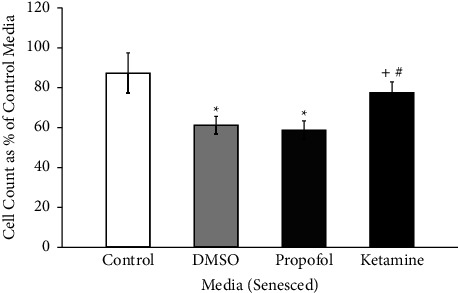
Cell counts of senesced astrocytes in DMSO, propofol, and ketamine. The average number of senescent astrocytes per exposure at all doses after seven hours of incubation in different media, expressed as a percentage of the best growth in control media for each set of experiments and presented as means, 95% confidence intervals. Control (*N* = 3; white bar), DMSO (*N* = 9 with low, medium, and high preparations included; light gray bar), propofol + DMSO (*N* = 9 with low, medium, and high preparations included; dark gray bar), and ketamine (*N* = 6 with low and high preparations included; closed bar). ^*∗*^*p* < 0.05 vs control; ^+^*p* < 0.05 vs DMSO; ^#^*p* < 0.05 vs propofol + DMSO. ANOVA with Bonferroni correction for post hoc comparisons.

Cell counts of senesced astrocytes were combined between doses of each assigned media. The effects of DMSO, propofol + DMSO, and ketamine on senesced primary culture human astrocytes are shown in Figure 5. When compared to the baseline control, there were significant reductions in total cell count in DMSO alone (*p* < 0.05) and in propofol dissolved in DMSO (*p* < 0.05). In addition, when compared to ketamine, there were also significant cell count reductions in DMSO alone (*p* < 0.05) and in propofol dissolved in DMSO (*p* < 0.05). These results indicate that the cell count reduction seen in the senesced group exposed to propofol emulsified in DMSO may not have been due to exposure to the anesthetic but rather to DMSO itself.

#### 3.3.3. Dose-Response Cell Count

**Figure 6:**
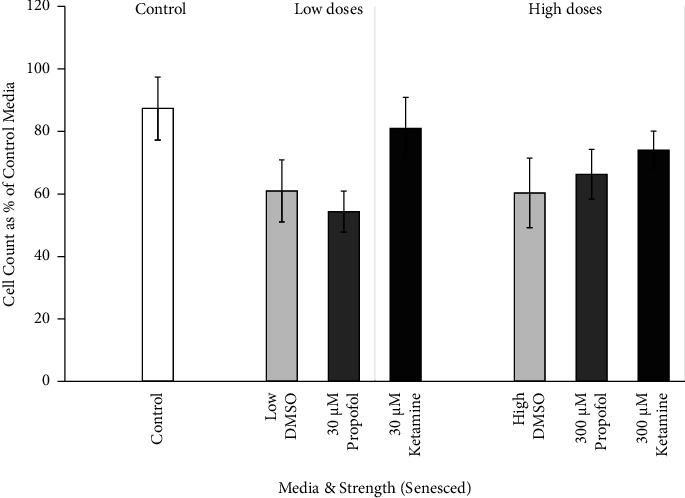
Cell count dose response of senesced astrocytes to DMSO, propofol, and ketamine. The average number of senescent astrocytes per dose after seven hours of incubation in escalating strengths of different media (30 *μ*M, low; 300 *μ*M, high; 100 *μ*M of DMSO and propofol + DMSO not shown), expressed as a percent of the best growth in control media for each set of experiments and presented as means, 95% confidence intervals. Control (*N* = 3; white bar), low DMSO corresponding to 74% DMSO used to prepare 30 *μ*M propofol (*N* = 3; light gray bar, left), high DMSO corresponding to 74% DMSO used to prepare 300 *μ*M propofol (*N* = 3; light gray bar, right), low propofol + DMSO (*N* = 3; 30 *μ*M dark gray bar, left), high propofol + DMSO (*N* = 3; 300 *μ*M dark gray bar, right), low ketamine (*N* = 3; 30 *μ*M closed bar, left), and high ketamine (*N* = 3; 300 *μ*M closed bar, right).

Dose-response cell counts of senesced astrocytes were examined between the low and high doses of each medium type. The effects of DMSO, propofol + DMSO, and ketamine on senesced primary culture human astrocytes are shown in Figure 6. There were no significant differences in cell counts between high-, medium-, and low-dose conditions of different medium incubations.

#### 3.3.4. Viability

**Figure 7:**
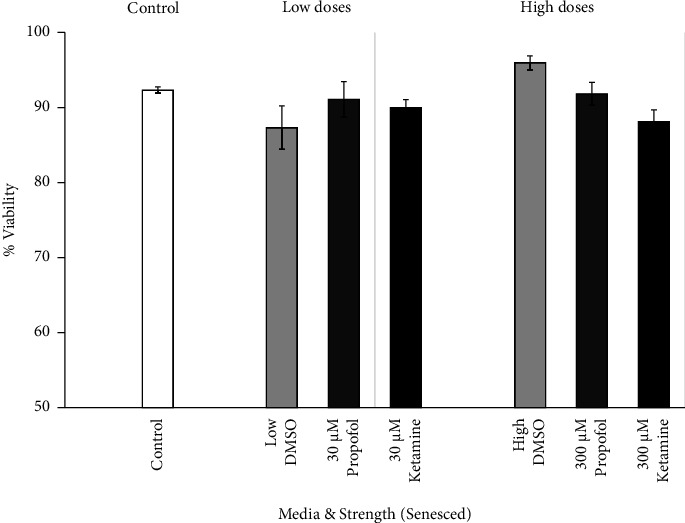
Viability dose response of senesced astrocytes to DMSO, propofol, and ketamine. The average percent viability of senescent astrocytes per dose after seven hours of incubation in escalating strengths of different media (30 *μ*M, low; 300 *μ*M, high; 100 *μ*M of DMSO and propofol + DMSO not shown), presented as means, 95% confidence intervals. Control (*N* = 3; white bar), low DMSO corresponding to 74% DMSO used to prepare 30 *μ*M propofol (*N* = 3; light gray bar, left), high DMSO corresponding to 74% DMSO used to prepare 300 *μ*M propofol (*N* = 3; light gray bar, right), low propofol + DMSO (*N* = 3; 30 *μ*M dark gray bar, left), high propofol + DMSO (*N* = 3; 300 *μ*M dark gray bar, right), low ketamine (*N* = 3; 30 *μ*M closed bar, left), and high ketamine (*N* = 3; 300 *μ*M closed bar, right).

The dose-response viability of senescent astrocytes was examined between the low and high doses of each medium type. The effects of DMSO, propofol + DMSO, and ketamine on senesced primary culture human astrocytes are shown in Figure 7. There were no significant differences in percent viability between the different medium incubation groups.

## 4. Discussion

Incubation of young astrocytes for seven hours in different medium types demonstrated a differential susceptibility of these cultures to certain commonly used anesthetics. Young astrocytes incubated in baseline control media grew to 375 ± 56 cells/aliquot. When the total cell counts among the different treatment groups were compared, astrocytes incubated with propofol dissolved in DMSO demonstrated a 39% reduction in cell count compared to the baseline control media. In contrast, young cells in propofol + DMSO had a 26% cell count reduction compared to DMSO alone and a 29% cell count reduction compared to ketamine. Interestingly, the effect of DMSO alone on primary cultures of young astrocytes was not as significant as that of propofol + DMSO, indicating a significant alteration of astrocytic survivability with exposure to this common anesthetic. These results contrast the effect of the anesthetics on senesced cultures of astrocytes, in which only total cell counts indicated a change in astrocytic survivability. Cultures exposed to DMSO alone and DMSO + propofol demonstrated a significant loss of cell count as opposed to the baseline control and ketamine. There was no dose-response effect of these anesthetics nor an alteration in our measures of viability. In no experimental group or condition did ketamine have any significant effect as compared to the baseline controls.

The growing understanding of the function of astrocytes intensifies the need to characterize the anesthesia effects on young and aged astrocytes. This goal is of particular significance as it impacts all populations, even beyond the extremes of age, as only about 30–50% of astrocytes are senescent in an adult [8]. Previous studies on the effect of aging on rat astrocytes suggest that these cells demonstrate declining function in culture that corresponds and is similar to observed changes in life, including characteristics of cellular senescence [25–27]. This observation may be why the results of the present study demonstrated a differential susceptibility of young, but not senesced, astrocytes to commonly used intravenous anesthetics when added to culture media, with ketamine showing a more favorable profile than propofol.

Propofol is frequently used for procedural sedation as well as for general anesthesia in children and adults. Propofol can act as a gamma-aminobutyric acid (GABA_A_) agonist [10]. Propofol has been shown to induce apoptosis in neurons, but its effect on astrocytes is less clear [28]. Ketamine is often used in infants and toddlers for elective procedures. This anesthetic is often utilized due to its short duration of action and use in procedures requiring dissociative amnesia with swift recovery time [29]. Ketamine may act as a noncompetitive blocker of *N*-methyl-d-aspartate (NMDA) receptor ion channels [29, 30]. Ketamine has been shown to induce apoptosis in cultured rat cortical neurons [31]. However, little is known about whether ketamine induces cell death in astrocytes.

Despite the widespread use of these agents, research on the effects of anesthetics on the brain has been scant and sometimes contradictory. This notion is complicated by the fact that several brain regions may be more vulnerable to anesthetics than others and thus may have differential impacts on cell populations within the nervous system [28, 32]. Multiple studies have found altered cognition and cellular development following anesthetic administration, particularly in vulnerable or developing populations [15, 16, 20, 23, 33].

Propofol has been found to have both neuroprotective and neurotoxic properties, depending on the conditions of the study, in a cell type, age, and time-dependent manner. Some studies have reported that propofol protects the brain [3, 24, 34] and have proposed propofol as an antioxidant agent that may protect neurons through regulation of oxidative stress, brain-derived neurotrophic factor (BDNF) pro-survival signaling, Na^+^/H^+^ exchange, and blood-brain barrier maintenance [24, 34, 35], as well as by reducing the effects of neuroinflammation on the nervous system [36]. Other studies have concluded that propofol may not significantly affect astrocytic viability, cell cycle, apoptosis, morphology, or the presence of reactive oxygen species [3, 28, 37, 38]. Conversely, propofol has been suspected to cause astrocytes to become activated and neurotoxic when exposed for more extended periods. It may also induce astrocyte apoptosis, as indicated by glial fibrillary acidic protein (GFAP) expression, cleaved caspase-3, cytokines, or other protein levels [20, 23, 39]. Recent research examining microRNAs has implicated propofol in increased expression of rno-miR-665, which preferentially binds and inhibits Bcl2l1, a significant anti-apoptotic protein [20, 23, 39]. Propofol has also been shown to change cerebral blood flow, alter astrocyte communication, decrease brainstem and cortical lactate release, increase neuronal calcium levels, impair memory and learning functions, and alter the electron transport chain in the mitochondria [16, 32, 40–45]. Our results indicate that propofol's effect on astrocytes may be more toxic. The data also indicated a strong dose-response of cell count in young primary cultures with exposure to propofol, over and above that of the DMSO vehicle alone.

The present study has focused on astrocytic cell count and viability as measures of astrocyte well-being. It is possible that other forms of viability may be impacted by these drugs that are not encapsulated in the Guava ViaCount assay that was conducted. However, the congruence between cell count and viability results signifies a detrimental effect of propofol on young astrocytes in culture.

The differential effect of these anesthetics on young and senesced cultures of astrocytes may be partly due to their mechanisms of action. The observed cell count reduction may be related to the adverse effects of propofol on mitochondrial function, as described in previous studies [45]. We also observed the detrimental effects of DMSO on young astrocytes in culture, compared to baseline controls. The findings on cell counts and viability by DMSO support the observed additional adverse effect of propofol on young astrocytes and its absence in cultures of senesced astrocytes as these cells have already been subjected to oxidative stress as part of their senescence process.

The reports on ketamine's effects are also controversial. Ketamine acts as a noncompetitive blocker of *N*-methyl-d-aspartate (NMDA) receptor ion channels [29]. Some studies have indicated ketamine's ability to induce reactive gliosis or astrocytic apoptosis [9, 46, 47] and neuronal apoptosis [33, 48]. Other studies have found that ketamine alone does not alter astrocyte survival or cognitive impairment following anesthetic administration [49]. Even further, some research has indicated that ketamine may also exhibit neuroprotective effects by increasing synaptogenesis, AMPA receptor expression, and arborization of dendrites [7]. Our study found no detrimental effects of ketamine on neither young nor senesced astrocytes in primary cultures.

It is important to note that while our results indicate that propofol may detrimentally impact young astrocytes, there are limitations to the applications of these data. For one, astrocytes in vivo are surrounded by a diverse network of other cells and environments. Our cells were not in any such framework. Additionally, we cannot conclude any clinical effect at this time due to this limitation.

We speculate that ketamine may have a more favorable safety profile than propofol in infants and young children. Further investigations should be conducted to translate this research into clinical settings and to elucidate the mechanism of anesthetic-astrocyte involvement.

## Data Availability

The data for this project are housed in the laboratory and are available upon request. Interested individuals should contact the corresponding author, Dr. Shadi Malaeb, at snm56@drexel.edu.
